# Effect of Stacking Sequence on Long-Term Creep Performance of Pultruded GFRP Composites

**DOI:** 10.3390/polym14194064

**Published:** 2022-09-28

**Authors:** Muhammad Rizal Muhammad Asyraf, Agusril Syamsir, Nazirul Mubin Zahari, Abu Bakar Mohd Supian, Fathoni Usman, Zarina Itam

**Affiliations:** 1 Institute of Energy Infrastructure, Universiti Tenaga Nasional, Jalan IKRAM-UNITEN, Kajang 43000, Selangor, Malaysia; 2 Engineering Design Research Group (EDRG), School of Mechanical Engineering, Faculty of Engineering, Universiti Teknologi Malaysia (UTM), Johor Bahru 81310, Johor, Malaysia; 3 Centre for Advanced Composite Materials (CACM), Universiti Teknologi Malaysia (UTM), Johor Bahru 81310, Johor, Malaysia; 4 Civil Engineering Department, Universiti Tenaga Nasional, Jalan IKRAM-UNITEN, Kajang 43000, Selangor, Malaysia

**Keywords:** stacking sequence, pultrusion, GFRP composite, creep properties, findley’s power law model, energy and transmission, electricity

## Abstract

Pultruded glass-fibre reinforced polymer (pGFRP) composites are classified as lightweight material, which exhibit high strength-to-weight ratio for structural usage. This composite material has been applied as cross-arm members in transmission towers due to its ability in thermal and electrical insulation. However, the influence of the stacking sequence of pGFRP composite on its mechanical performance has not been fully covered in the literature to explain the long-term durability of the current cross-arm designs. The study expected to evaluate five fiber layers with various stacking sequences in terms of quasi-static and creep tests in a four-point bending mode. The creep test was performed for 1440 h (60 days). These composites were fabricated using the pultrusion process in the form of a square hollow structure. Later, it was cut into composite coupons with various sizes depending on the test conducted. The results showed that nine layers with 0°/45°/0°/−45°/0°/−45°/0°/45°/0° had the ultimate flexural strength. This stacking sequence configurations seemed to be optimally manufactured in continuous roving fibre by alternating between 0° and ±45° fiber orientations. Additionally, the S-9 pGFRP composite sample showed that it had a low-creep deflection with high elastic and apparent creep moduli in 1440 h. In terms of strength reduction factor, this configuration was recorded as the highest. The findings showed that the nine layers of pGFRP composites with alternation of 0° and ±45° fiber orientations were highly suitable for structural application at transmission towers for a long-term operation.

## 1. Introduction

The fibre reinforced polymer (FRP) composites have been chosen by engineers in high-performance product applications due to their tremendous properties. The demand of FRP composites is increasing in various sectors due to its lightweight property, high stiffness-to-weight and strength-to-weight ratios, ease of installation, and potentially high overall durability [1,2]. In general, there is a wide range of civil and structural engineering uses which employ the FRP composite such as a reinforcement material in strengthening of walls [3,4,5], reinforcing rods and tendons [6,7], FRP composite structural systems [8,9,10], wraps for seismic retrofit of columns [11], and composite bridge decks [12,13,14]. From this point of view, the properties of FRP composites are deliberated by most engineers due to its ability to perform under extreme environmental conditions [15]. Among all, pultruded glass fibre-reinforced polymer (pGFRP) composites have been identified as low cost and have a high strength-to-weight ratio, which is mainly used in automotive, aerospace, civil, and defence applications [16,17,18,19,20].

The pGFRP composites have been implemented in various applications including cross arms in transmission towers [21,22,23] and pipeline systems [24,25,26,27]. Cross arms in suspension latticed transmission towers have used the pGFRP composite due to the replacement of the old wooden timber member [28,29]. The pGFRP composites are known to be distinguished in terms of high mechanical strength, electrical and thermal insulations, and well performance in terms of dielectric strength [30,31]. This mitigation step was conducted by an electrical provider corporation due to the drastic decrease of the wood timber sources and failure issues of wood in long-term applications [32,33]. Creep is a critical issue in shortening the lifespan of the cross arms besides the exposure to extreme climate conditions and biological attacks [34]. It can be justified that the pGFRP composites are among the most appropriate alternative materials to replace wooden cross arms in transmission towers [35,36]. The combined factors between good electrical properties and the issues with wooden cross arms have become the drive factors to implement pGFRP composite as a primary material in cross-arm assembly.

Creep is a state where a material experiences a trend of deformation under long-term constant applied stresses. The creep deformation patterns are exhibited after instantaneous deformation and consist of three stages; primary (transient), secondary (steady-state), and tertiary (accelerated). During the primary stage, the deformation pattern shows a decreasing trend at function of time due to strain hardening [37]. Prior to this phase, the second stage displays the creep rate that remains almost constant when the state of balance is attained between the rate of recovery and the dislocation generation rate [38]. The tertiary stage happens after a creep steady-state condition, and it can be observed that creep rate rapidly escalates until the material breaks [39]. Hence, it can be justified that long-term creep responses would lead to several material defects such as shear yielding, polymer chain slippage, fibre breakage, void creation, and growth [40,41]. At the end, the occurrence of creep would cause the material to fail instantaneously without any sign. Currently, the pGFRP composite cross arms used in transmission towers are considered anisotropic material as it is made up of E-glass fibre reinforced unsaturated polyester composite by the pultrusion process [42]. This polymer composite may have the potential to exhibit a lack of instability of fibre interfacial strength, which subsequently contributes to faster creep rate.

To solve this issue, several research works have been proposed to implement the optimal stacking sequence of fibre to allow better stress transfer between fibre and matrix [43,44,45]. In general, the mechanical performance of polymer composites can be optimized by considering layering the sequence and fibre orientation with other factors such as interphase matrix and fibre volume [46,47,48,49,50]. To be specific, the stacking sequence of fibres of polymer composites plays a vital role to enhance the mechanical properties of composite structures especially on a long-term performance. Previous studies show that woven textiles are effective reinforcing structures in facilitating physical interferences within reinforced fibre and matrix systems [51,52,53]. Various literature works have been written on fibre reinforced polymer composites on quasi-static mechanical and failure mode responses [54,55,56,57,58]. However, no experimental study has been introduced to evaluate the long-term performance of pGFRP composite with the optimal stacking sequence. Currently, most studies on pGFRP composite cross arms involve computational simulation such as the impact of variation magnitude static load [54]; the influence of the laminating sequence of composite cross arm assemblies [59], and the failure properties of cross-arm structures under multi-axial load with distinct laminate properties [60]. Despite the research conducted on the numerical simulations of composite cross arms, experimental studies on the creep performance of pGFRP composite cross arms remain lacking.

The goals of this research is to describe and compare the variation of fibre stacking sequences on quasi-static flexural and creep performance of pGFRP composites. Generally, the pGFRP composite possesses great mechanical strength to resist high load concentration for structural usage [61,62]. Despite many studies focusing on the investigations of FRP composites’ stacking sequences, a comparative study on the effects of stacking sequence on long-term mechanical performance of pGFRP cross arms has still not been fully conducted. This study aims to analyse the quasi-static and creep properties of five distinct fibre stacking sequences of pGFRP composites under a 4-point bending mode. The experiment commenced by using three load levels from the ultimate flexural strength to obtain the creep properties along the duration of 1440 h (60 days). Additionally, this work intends to create a baseline knowledge of pGFRP composite cross arms under various fibre sequences and orientations. Hence, the outputs of this research are expected to deliver a practical viewpoint to structural engineers and material scientists in extending the knowledge of the long-term properties for pGFRP composite cross arms.

## 2. Material and Methods

### 2.1. Fabrication Process and Material Properties

**Figure 1:**
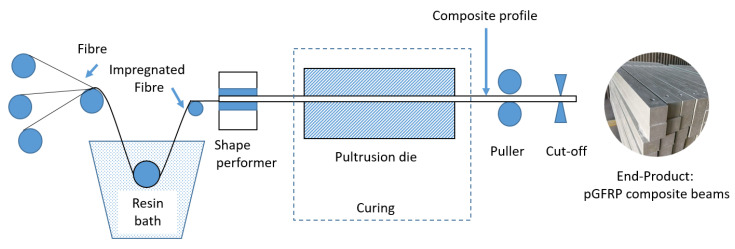
Schematic diagram of pultrusion process of glass fibre/UPE composite beams.

Pultrusion is a manufacturing technique which involves a pull and extrusion mechanism to form a symmetric cross-section shape of composite beams. This research assessed the long-term mechanical properties of pGFRP composite beams fabricated via pultrusion. The pGFRP composite samples were manufactured from a multi-dimensional E-glass fibre reinforced unsaturated polyester (UPE) resin to sustain the high load capacity for structural applications [63,64]. During the pultrusion process as shown in Figure 1, the fibre strand was pulled out from the resin bath of liquid thermosetting UPE through the curing die to form a square hollow shape of solid composite. The resin that impregnated the uni-directional fibre strand was encrusted together with a fibre mat in sequence inside the die. Finally, pGFRP composite beams were ready to be used as cross-arm members in transmission towers. In this project, the composite samples were obtained directly from local pultrusion manufacturers for pultruded composite cross arms. These composites follow the actual standard physical size and dimensions of the industrial requirements and specifications as proposed by the Tenaga Nasional Berhad (TNB). Each composite cross-arm beam was cut from the wall segments of the square hollow composite beams with a thickness of 7.0 ± 0.2 mm and equal height and length of 102 × 102 mm^2^. Generally, each thickness layer of the pGFRP composite is in between 0.72 and 3.60 mm depending on the number of layers [60].

**Figure 2:**
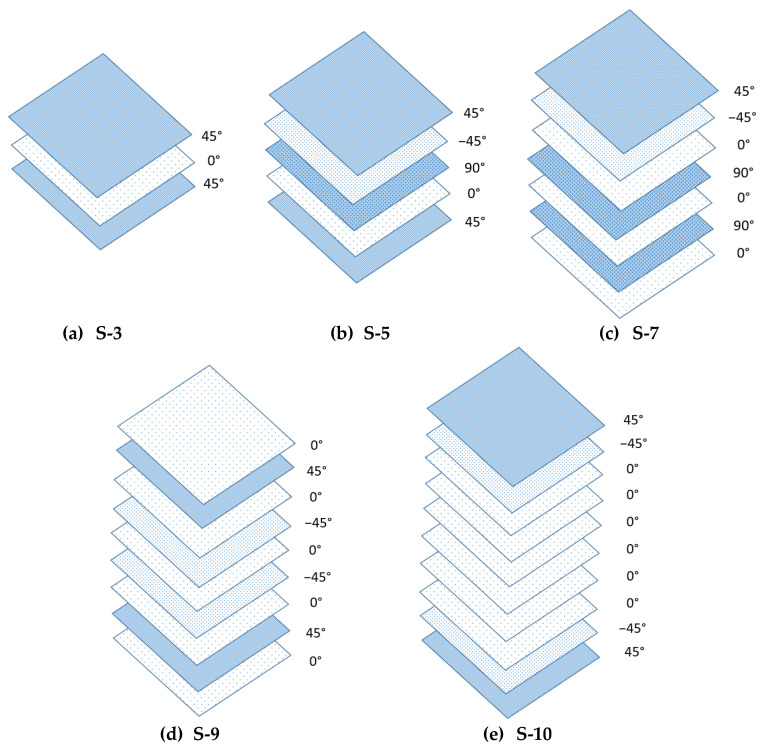
Stacking sequence of pGFRP composite samples such as (**a**) S-3, (**b**) S-5, (**c**) S-7, (**d**) S-9 and (**e**) S-10.

Figure 2 displays the stacking sequences (3, 5, 7, 9, and 10) with various fibre arrangements of the composite beams. The samples were later cut in coupons to be tested for quasi-static and creep tests. Table 1 displays the details of five different stacking sequences with various fibre arrangements of pGFRP composite.

Nine replications were prepared for each composite configuration to be applied for the quasi-static flexural and flexural creep tests. Table 2 illustrates the details of mass percentage of fibre and resins for each stacking sequence. The details for each test are elaborated in specific sections.

### 2.2. Quasi-Static Flexural Test

**Figure 3:**
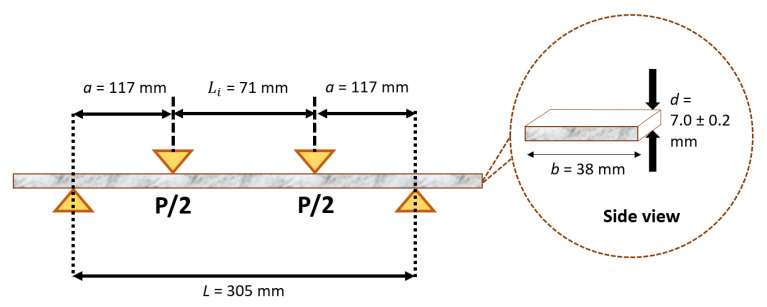
Schematic diagram of flexural creep setup as per ASTM D6272 with dimensions of flexural specimens.

The flexural test was executed using a 4-point bending method in accordance with the international testing standard, ASTM D6272, as shown in Figure 3. This is to ensure that the sample is subjected to constant flexural stress at the sample midpoint. The test was conducted using a Universal Testing Machine (Instron, Norwood, Massachusetts, USA) at the Material Laboratory, Universiti Tenaga Nasional, Malaysia. This experiment was expected to evaluate the ultimate flexural strength with a crosshead speed of 3.29 mm/min. The diameter of the loading channel was set at 2.5 mm with a support span length of 305 mm and 12.7 mm of diameter support roller. The setup of the experiment involved the span-to-depth support ratio of 29 with the span-to-depth shear ratio being approximately 10.45. This technique was employed since it was compatible with the ASTM D6272 criteria and slender enough to decrease shear effects. The dimension of the specimen was set at 320 mm × 38 mm, and each sample configuration was replicated three times. The sample thickness was 7.0 ± 0.2 mm. The end results were averaged and recorded for analysis and discussion. The ultimate flexural strength of pGFRP composites was calculated using Equation (1):

(1) $$\sigma =\frac{3P\left(L-Li\right)}{bd^{2}}$$

where *σ* is applied stress, *P* is load at a given point on the load-deflection curve, *L_i_* is length of loading pin, *L* is support span, *b* is width of beam, and *d* is depth of beam.

### 2.3. Flexural Creep Test

**Figure 4:**
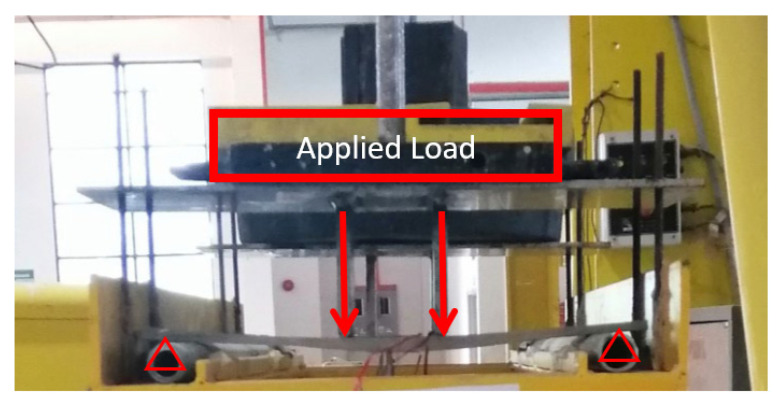
A coupon sample under four-point bending creep.

Flexural creep test was arranged in the same setup as in the quasi-static flexural test as shown in Figure 4, with equivalent dimensions for loading and support spans. The test employed a 4-point bending technique to evaluate the creep performance in a period of 60 days (1440 h operation). This test followed the international standard of creep test for polymer composite, ASTM D2990. The test was conducted at the Laboratory of Civil Engineering, Universiti Tenaga Nasional, Malaysia, using a custom-made coupon creep test rig. Three load levels were established, such as 12%, 24%, and 37%, of the ultimate flexural load. The experiment was applied in an enclosed area at room temperature for 1440 h. The 1440 h of creep operation was selected to allow creep to progress to the second stage. During test operation, mid-span deflection was logged right after loading; at 0 s, and then the deflection outputs from dial gauge were recorded at 1, 6, 12, and 30 min; 1, 2, 5, 20, 50, 100, 200, 500, 700, 1000, 1200, and 1440 h. A displacement transducer dial gauge was used to record the deflection at the midspan of the sample for various time periods, ranging from 15 min to 1440 h.

### 2.4. Numerical Analysis: Findley Model and Creep Elastic Modulus

The analysis of creep performance can be evaluated by implementing an established creep model, which is a Findley model. This model is also known as a nonlinear numerical model to justify creep strain trends in terms of stress factor and material constants in the long term [65,66]. Technically, the model is useful to eliminate any exaggerated data deviation since it is considered as a simple and straightforward model. The Findley power law model was used to forecast the long-term mechanical performance of anisotropic material, such as the pGFRP composite. The computer software used to attain the nonlinear fitting function of the Findley model is Origin Pro 2016. To organise and analyse the creep data from the experimental findings, the creep strain versus time at three different stress values were installed in the computer software. The fitting was done by incorporating Equation (2) in the mentioned modelling software. In this case, the mean values of *m* and *n* can be computed in the numerical analysis. It can be derived by using the least-square curve fitting of the results of the flexural creep test:

(2) $$\varepsilon \left(t\right)=\varepsilon_{0}+mt^{n}$$

where *ε*_0_ is the instantaneous time-independent strain and *m* is the stress-dependent, and *n* is a stress-independent material constant. Findley’s numerical method was engaged to analyse the time-dependent reduction factor [67,68]. In this case, the reduction factor was used to assess the reduction in modulus of elasticity (creep modulus) using the *n* and *m* parameters for each sequence. 

The time-dependent reduction factor can be calculated using the following equation:

(3) $$\chi \left(t\right)={\left(1+\frac{E_{0}}{E_{t}}*t^{n}\right)}^{-1}\;$$

where *E*_0_ is the material (initial) elastic modulus, *E_t_* is an apparent constant modulus for creep, *t* is the time operation, and *n* is the stress-independent material constant. Thus, the new elasticity modulus is expressed as follows:

(4) $$E\left(t\right)=E_{0}\;*\chi \left(t\right)\;$$

where χ(*t*) is the time-dependent reduction factor.

## 3. Results and Discussion

### 3.1. Ultimate Flexural Strength

A 4-point flexural test was carried out to evaluate the flexural strength of different stacking sequences of pGFRP composites. As previously discussed, short-term flexural strength results are essential to govern the load percentages for the creep tests, which are needed to obtain the ultimate flexural strength. Table 3 displays the maximum flexural load and ultimate flexural strength for each pGFRP composite configuration. From Table 3, it can be noticed that the maximum flexural load of three sample tests for all composite configurations were closed to each other, which directly indicated the precision and accuracy of the data. In terms of percentage errors, it can be found that all pGFRP configurations had percentage errors below 3%, which showed that the results were highly accurate. Generally, the percentage errors to validate the experimental data were classified to several classes, such as unsatisfactory, which is more than 25% error, fair, which is between 20 and 24.9% error, satisfactory at 15 to 19.9% error, good which is in between 10 and 14.9% of error, and highly acceptable, which was 0.1 to 9.9% error [69,70].

**Figure 5:**
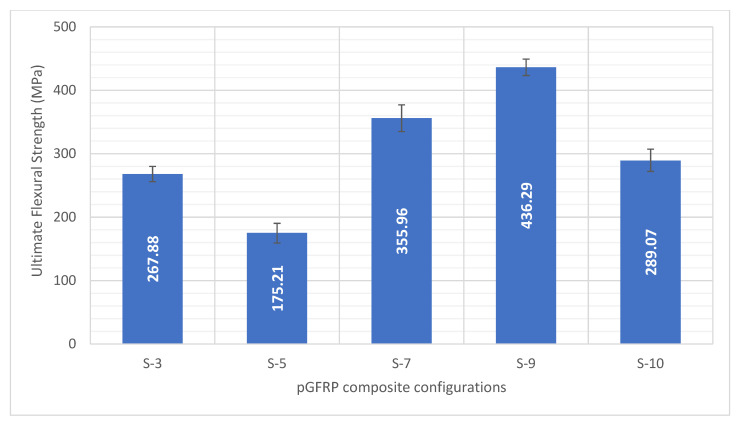
Ultimate flexural strength of pGRRP composites with various stacking sequences.

In this experiment, the ultimate flexural strengths of S-3, S-5, S-7, S-9, and S-10 of pGFRP composites were evaluated. In Figure 5, the flexural strength of S-3 to S-5 pGFRP composites decreased, but at S-5 to S-9 configurations, the flexural strengths increased to the maximum, which was 289.07 MPa (S-9 pGFRP composite). Based on the results, the highest flexural load was seen at S-9 sample at 2759 N, while the lowest value of flexural load was recorded by S-5 sample. In conjunction with these results, the highest ultimate flexural strength recorded was S-9 (436.29 MPa) as shown in Figure 5. 

These results were due to higher content of continuous roving in fabricating the profiles since the lamination sequence was alternated between 0° and ±45° [71,72]. Additionally, both sides of the 0° outer layers of pultruded composite allowed principal loading direction which was aligned with the ply orientation according to Hashim et al. [73]. Thus, this condition would allow the pultruded composite to experience an orthogonal manner to increase the flexural strength of the composite [74]. Additionally, the alternation between 0° and ±45° of the ply orientation resulted in varied stiffness along the thickness of the laminates [75,76,77]. These findings also showed the layering number did not give a significant improvement in the mechanical properties of pGFRP composite since the S-10 configuration with ten layers of fibres had lower flexural strength (289.07 MPa) than S-9 configuration (436.29 MPa).

### 3.2. Flexural Creep Properties

**Figure 6:**
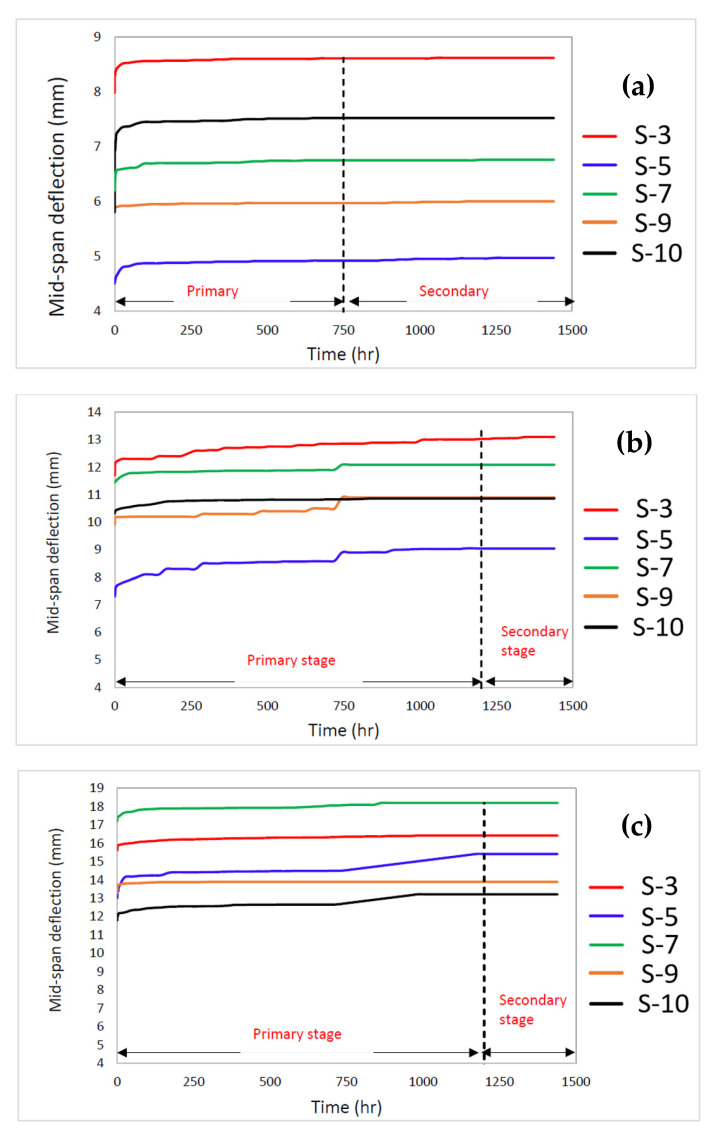
Creep deflection for pGFRP cross arm that subjected to (**a**) 12%; (**b**) 24%; and (**c**) 37% of load levels.

The results of the 4-point flexural creep test are presented and elaborated in this subsection. Table 4 defines the maximum flexural load and creep load levels for individual stacking sequences. The creep load levels were calculated based on the average maximum flexural load obtained in a quasi-static flexural test. Figure 6 displays the comparison of the creep deflection-time curve for five pGFRP composite configurations.

The creep trends from Figure 6 display the same creep pattern as in previous works, which evaluate the impact of stacking sequence on its long-term performance of pGFRP laminates [78,79]. The creep trend of pGFRP composites was affected by the stacking sequence and caused the results to have large differences in these configurations. Additionally, the results showed that more stacking sequences in composite laminates suggested that higher fibre volume fraction contributed towards lowering its resistance of applied force. The less resin usage could be a reason to be reduce creep deformation in pGFRP composites, especially in the primary stage [78]. This finding emphasizes the significance of having higher fibre volume percentage, which allows increase in the composite structure’s strength, stiffness, and resistance to creep.

Based on the findings, it can be clearly seen that all five pGFRP composites’ curves experienced two creep stages, which are primary (transient) and secondary (steady-state) stages. The primary creep allowed the anisotropic material to experience high elasticity behaviour, while the secondary creep caused the material to be viscoelastic before entering plastic regions [80,81,82]. At the load level of 12%, the primary stage from 0 to 750 h, the secondary stage started at 750 h of loading. At the load level of 24% and 37%, the secondary stage started after 1200 h of loading. The transition between primary and secondary creep phases seemed different for these three load levels. The 12% load permit showed shorter time in transitioning between these phases. The decreasing creep rate in the primary creep stage has been attributed to strain hardening or reduction in the density of free or mobile dislocations [83,84,85]. 

**Figure 7:**
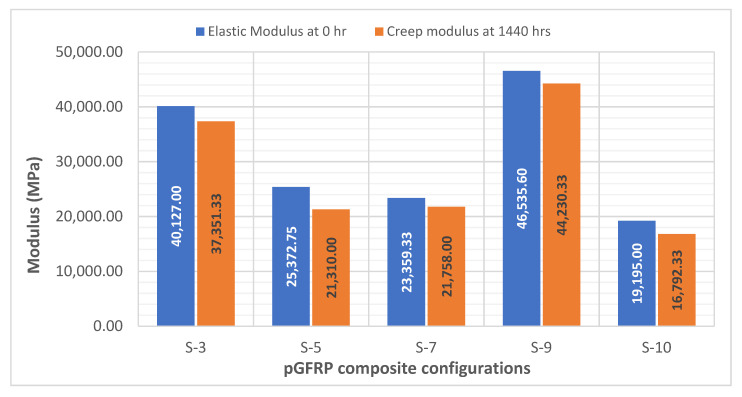
Changes of creep modulus between initial and final periods for all pGFRP composite configurations.

Figure 6a shows that the initial deflection of S-5 sample is the lowest deflection compared to others at the 12% load level. This was probably due to the S-5 sample which exhibited the lowest ultimate flexural strength among all other samples as displayed in Figure 5. For Figure 6b,c, S-3 and S-7 are the highest deflection for 24% and 37% load levels, respectively. The inconsistency on the deflection of the sample may be due to different thickness of each configuration as well as the number of fibre layers. Besides that, the results illustrated that the S-9 sample had lesser deflection increment in comparison with other pGFRP composite samples. It can be deduced that the ability of the S-9 sample can resist the sustained load without additional deflection, which indicated that it probably had a longer lifespan. According to Fotuohi et al. [75], the alternation between 0° and ±45° of the ply orientation results in varied stiffness along the thickness of the laminates, which causes the S-9 sample to have better less creep strain rate. In this case, an optimised layering sequence plays a significant role towards high performance of structural applications [44,45,86]. At the end of this study, the secondary stage at 1440 h was selected in determining Findley’s parameter as well as the reduction factor.

### 3.3. Creep Analysis Using Findley’s Power Law Model

As mentioned in the previous section, the creep results were evaluated using the Findley’s power law model to identify the creep modulus and strength reduction along 1440 h. Initially, the *m* and *n* values were obtained from creep data simulated using Equation (2) as displayed in Table 5. 

These values were used to calculate the reduction factor, (*χ*(*t*)), using Equation (4). Based on these strains, the elastic modulus (*E*) of the samples after 1440 h was calculated. The final step of this calculation was to find the reduction factor of the elastic modulus (*χ*(*t*)). Table 6 displays the creep data obtained based on the Findley’s power law model for all composite configurations. These parameters include the instantaneous deflection (*ε*), elastic modulus (*E*_0_), apparent creep modulus (*E_t_*), and strength reduction factor (*χ*(*t*)). As displayed in Table 6, the elastic modulus of pGFRP composite with various stacking sequences exhibits a drastic change in value. The S-5 sample had the highest degradation value of creep modulus after 1440 h as the strength reduction factors are in the range of 0.792 to 0.891. This was due to its stacking sequence of 45°/−45°/90°/0°/45°. The S-5 sequences were fabricated using layers of chopped strand mat glass fibre and twill glass cloth. This layering condition could not enhance the stiffness and strength of the composites [61,87].

As shown in Figure 7, the most noteworthy modulus reduction after 1440 h was an S-5 sample with a reduction percentage of 84% (0.84 of strength reduction factor). Meanwhile, the least reduction percentage of initial and final creep modulus was the S-9 sample.

**Figure 8:**
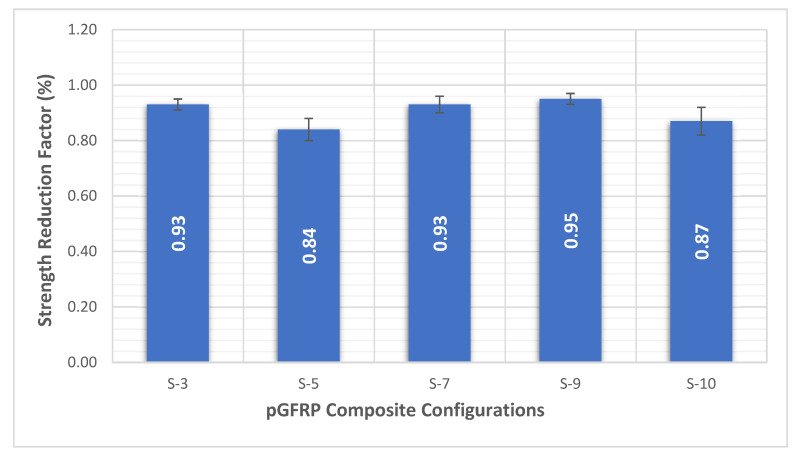
Average strength reduction factor in 1440 h (60 days).

Figure 8 shows that the S-3, S-5, S-7, S-9, and S-10 samples have reduction factors of 0.93, 0.84, 0.93, 0.95, and 0.87, respectively. It was found that the S-9 sample had the highest strength reduction factor of 0.95. This finding illustrated that the S-9 configuration pGFRP composite had effectively enhanced the strength of the pGFRP composite in a long-term application. The application of 0° at both outermost layers would provide load distributors, which distribute the force uniformly within the polymer matrix [88,89]. The middle alternation orientation between ±45°/0° sequences aided as an energy absorber [76,90,91]. Amaro et al. [92] and Reis et al. [93] established that the stacking sequences with variation of thickness of ply layer significantly contribute to the enhancement of energy absorbing properties for polymer composites. Besides that, Morioka and Tomita [94] establish that the stacking sequence of fibre with +45° direction allows higher energy absorption and emits slow bends which contribute to betterment modulus. Thus, the application of the S-9 pGFRP composite can significantly increase the bending stiffness in a continuous load application for a long-period service.

## 4. Conclusions

This study focuses on the evaluation of a long-term mechanical performance of pGFRP composite with various stacking sequences. An experiment is done to carry out the investigation of flexural creep performance of glass fibre reinforced polymer laminates made by pultrusion with variation stacking sequences. Five stacking sequences are used to evaluate the short- and long-term mechanical properties of the pGFRP composites. These experiments were conducted using four-point flexural configurations and dimensions. The obtained creep data were analysed using Findley’s power law model to achieve the reduction factor, χ(*t*), and time-dependent modulus, *E*(*t*). Overall, it can be deduced that S-9 with a stacking sequence of 0°/45°/0°/−45°/0°/−45°/0°/45°/0° sample has the most superior performance compared to others in terms of ultimate flexural properties. This is due to the influence of stacking sequence arrangement of fibre alternating between 0°/±45°, which gives good strength for structural usage. Additionally, it can be clearly seen that the number of fibre layering would not contribute much to the overall bending strength. In terms of flexural creep properties, the secondary stage (steady-state) creep of pGFRP composite would happen between 750 h and 1200 h depending on the load applied. The inconsistency on the deflection of the sample may be due to each configuration having different thicknesses as well number of fibre layers. The pGFRP composite with 0°/45°/0°/−45°/0°/−45°/0°/45°/0° has low creep deflection due to the influence of its high flexural strength. For Findley’s model analysis, it can be concluded the S-9 configuration has the highest elastic and apparent creep moduli compared to others. Additionally, the strength reduction factor of this configuration is the highest among all samples which shows that this configuration exhibits an optimum stacking sequence for structural applications.

Generally, Findley’s power law model is a useful tool to elaborate and justify the long-term creep responses and performances of pGFRP composites in duration more than 1000 h. This numerical analysis technique could forecast the pGFRPC cross arms at low creep rates within the normal working loads. It is recommended to carry out the creep experiments of pGFRP composites under the influence of UV rays, humidity, and temperature. This study is essential to further extend the knowledge of the long-term creep behaviour of the composites, especially for the applications of composite cross arms on transmission tower. Technically, all composite cross arms in the transmission lines are operated in an outdoor environment, which means exposed to extreme tropical climate. Thus, the suggested work could broaden the views and perspectives of engineers and scientists on pGFRP composites for cross-arm structure applications in transmission towers.

## Figures and Tables

**Table 1:** Configuration of glass fibre/UPE composites.

No.	Configuration	Number of Layer	Layering Sequence
1.	S-3	3	45°/0°/45°
2.	S-5	5	45°/−45°/90°/0°/45°
3.	S-7	7	45°/−45°/0°/90°/0°/90°/0°
4.	S-9	9	0°/45°/0°/−45°/0°/−45°/0°/45°/0°
5.	S-10	10	45°/−45°/0°/0°/0°/0°/0°/0°/−45°/45°

**Table 2:** Fibre and polymer fraction mass for each configuration.

No.	Configuration	Fibre Mass (%)	Resin Mass (%)
1.	S-3	64.11	35.89
2.	S-5	64.67	35.33
3.	S-7	72.77	27.33
4.	S-9	78.04	21.96
5.	S-10	74.17	25.83

**Table 3:** Maximum flexural load and ultimate flexural strength of pGFRP composites.

pGFRP Composite Configurations	Sample	Maximum Flexural Load (N)	Average Maximum Flexural Load (N)	Standard Deviation	Percentage Error (%)	Ultimate Flexural Strength (MPa)
S-3	Sample 1	1697.38	1694	24	1.42	267.88
	Sample 2	1724.78				
	Sample 3	1704.73				
S-5	Sample 1	1152.99	1108	31	2.94	175.21
	Sample 2	1051.14				
	Sample 3	1091.80				
S-7	Sample 1	2250.65	2251	42	1.07	355.96
	Sample 2	2251.88				
	Sample 3	2281.96				
S-9	Sample 1	2799.12	2759	26	0.94	436.29
	Sample 2	2765.66				
	Sample 3	2783.42				
S-10	Sample 1	1789.24	1828	37	2.02	289.07
	Sample 2	1820.41				
	Sample 3	1868.92				

**Table 4:** Summary of the maximum flexural load and creep load levels.

Sequence	Ultimate Flexural load (N)	Load Level	Applied Load (N)
S-3	1694	12%	203.28
		24%	406.56
		37%	626.78
S-5	1108	12%	132.96
		24%	256.92
		37%	409.96
S-7	2251	12%	270.12
		24%	540.24
		37%	832.87
S-9	2759	12%	331.08
		24%	662.16
		37%	1020.83
S-10	1694	12%	203.28
		24%	406.56
		37%	626.78

**Table 5:** *m* and *n* values for all composite configurations.

Samples	Load (%)	*n*	Average *n*	*m*	Average *m*
S-3	12	0.0954	0.1769	0.0097	0.00743
	24	0.2315		0.0071	
	37	0.2038		0.0055	
S-5	12	0.3000	0.3070	0.0020	0.00567
	24	0.3424		0.0051	
	37	0.2786		0.0099	
S-7	12	0.1290	0.2694	0.0088	0.00553
	24	0.4340		0.0012	
	37	0.2452		0.0066	
S-9	12	0.149	0.1317	0.0019	0.00737
	24	0.1803		0.0071	
	37	0.0658		0.0131	
S-10	12	0.0582	0.1920	0.0464	0.01953
	24	0.2843		0.0031	
	37	0.2335		0.0091	

**Table 6:** Flexural creep data based on Findley’s power law model.

Configurations	Load Level (%)	Instantaneous Deflection, *ε* (mm)	Elastic Modulus, *E*_0_ (MPa)	Creep Modulus at 1440 Hrs, *E_t_* (MPa)	Strength Reduction Factor, χ(t)
S-3	12	2.20	30,543.00	28,232.00	0.942
	24	3.30	41,664.00	37,874.00	0.909
	37	4.30	48,174.00	45,948.00	0.954
S-5	12	1.96	23,064.85	20,544.00	0.891
	24	3.34	28,436.11	22,523.00	0.792
	37	4.48	24,617.29	20,863.00	0.847
S-7	12	2.32	21,930.00	19,994.00	0.910
	24	4.28	23,771.00	22,304.00	0.940
	37	6.44	24,377.00	22,976.00	0.940
S-9	12	1.96	39,556.02	38,457.00	0.970
	24	3.34	46,588.20	43,179.00	0.930
	37	4.48	53,462.59	51,055.00	0.950
S-10	12	2.20	15,812.00	12,004.00	0.760
	24	4.00	17,790.00	16,756.00	0.940
	37	4.50	23,983.00	21,617.00	0.900

## Data Availability

The data used to support the findings of this study are included within the article.
